# Trends of electronic cigarette use among adolescents: A bibliometric analysis

**DOI:** 10.18332/tid/191761

**Published:** 2024-08-22

**Authors:** Wenqi Chen, Gaoran Chen, Shaojie Qi, Jinzheng Han

**Affiliations:** 1 Research Institute of Social Development, Southwestern University of Finance and Economics, Chengdu, China

**Keywords:** electronic cigarette, adolescents, bibliometric analysis

## Abstract

**INTRODUCTION:**

The use rate of electronic cigarettes (e-cigarettes) among adolescents is continuously rising globally, posing new challenges to public health and negatively impacting adolescent health. This study employs bibliometric methods to systematically present the current state and evolving trends in global research on adolescent e-cigarette use.

**METHODS:**

This study uses CiteSpace to conduct a bibliometric analysis of articles related to adolescent e-cigarette use from the Web of Science (WoS) Core Collection database. Firstly, performance analysis and collaboration network analysis were utilized to clarify the basic publication status, main knowledge producers, and knowledge collaboration networks in adolescent e-cigarette use research. Secondly, a co-citation network analysis was performed to visually analyze the disciplinary characteristics and ‘hot topics’ in this field. Finally, keyword burst detection and clustering techniques were employed to further explain the development trends and frontiers of research on adolescent e-cigarette use.

**RESULTS:**

A total of 2063 research articles and review articles were included in this study. Research on adolescent e-cigarette use has significantly increased from 2002 to 2024. The United States, the United Kingdom and Canada are the main contributors, with their institutions and researchers playing key roles in the international collaborative network. Current research increasingly adopts interdisciplinary approaches. Keyword co-occurrence and burst identified current research ‘hotspots’ including vaping, substance use, public policy, prevention, advertising, and cessation. Co-citation cluster analysis revealed promising research areas such as attractiveness, environment and health, accessibility and smoking behavior, and mental health.

**CONCLUSIONS:**

Through data mining and visualization techniques, this study provides a comprehensive bibliometric analysis of published work on e-cigarettes and adolescence. The results of this work offer references for researchers in future investigations.

## INTRODUCTION

The worldwide use of electronic cigarettes (e-cigarettes) is becoming increasingly prevalent, particularly among adolescents^1^. According to the 2023 National Youth Tobacco Survey (2023), 10% of US high school students reported using e-cigarettes in the past 30 days, which is approximately five times the rate of traditional cigarettes use^2^. This escalating trend is alarming and could present new health challenges for adolescent development.

Electronic cigarettes, also known as vaping products, are electronic devices designed to simulate the experience of traditional tobacco products, providing users with a variety of designs and flavors. The expansion of the e-cigarette market and the prevalence of online advertising have provided teenagers with increased access to these products through various channels^3,4^, thereby heightening their exposure to the adverse health outcomes and risks associated with their use, including oral health issues, respiratory problems, as well as substance use and nicotine dependence^5^. The rate of e-cigarette use among children and adolescents is growing alarmingly in many countries. Across all WHO regions, e-cigarette use among those aged 13–15 years exceeds that of adults^6^. For instance, in Canada, e-cigarette use among teenagers aged 16–19 years doubled from 2017 to 2022, and in England, the number of young users doubled over the past three years^7^.

The use of any tobacco product is unsafe for children and adolescents^8^. Most e-cigarettes contain nicotine and other harmful chemicals, which can potentially lead to lung damage, cardiovascular diseases, brain disorders and mental health issues^9-11^. Even products labeled as nicotine-free cannot escape these risks, some zero-nicotine e-cigarettes have been found to contain nicotine^12^.

A considerable amount of research, including several systematic reviews, have been conducted to investigate the motivations, associations, and consequences of their use^12-14^. However, these studies often have a narrow focus and do not provide a comprehensive, systematic examination of the development trajectory of adolescent e-cigarette research, necessitating further analysis to fully and impartially explore the knowledge production processes and thematic trends in this field.

In this study, a scientometric analysis is performed to map the knowledge structure and trends in adolescent e-cigarette use, constructed on the basis of a large bibliometric database. Specifically, several important questions will be answered: 1) ‘What are the overall changes and developments in this field?’; 2) ‘Which countries, institutions and authors are most productive and influential?’; and 3) ‘What are the past and current research hotspots in the field, and how will future trends evolve?’. Answers to these questions hold significant implications for both academic and applied research concerning e-cigarettes for adolescents.

## METHODS

### Data collection

This study focuses on adolescents and e-cigarettes, sourcing relevant articles from the Web of Science (WoS) Core Collection databases. WoS, housing many leading academic journals, is known for its systematic and rigorous selection criteria, authoritative data, stable information, and extensive resources, making it a reliable choice for researchers^15,16^. Considering the complex implications of electronic cigarettes, the study employs both computerized searches and manual screening for data collection.

The search string included: [(Electronic-Cigarette* OR e-cigarette* OR e-cig* OR e-liquid OR e-juice OR e-hookah OR Vape OR vaping OR vaper OR electronic-nicotine-delivery-system OR electronic-non-nicotine-delivery) OR (electronic and cigalike OR mods OR tobacco OR nicotine OR hookah OR vapori*)] AND [child* OR teenager OR adolescent*]. Search in articles included the title, abstract, and keywords of articles. Subsequently, the search was refined with criteria specifying: document type = article + review; and language = English. All publication searches were completed 31 March 2024, with initial findings totaling 2978 records after limiting document types and languages. Additionally, two researchers simultaneously conducted the screening (Supplementary file Figure 1), adhering to the PRISMA statement guidelines^17^. After excluding duplicates, irrelevant publications, and articles missing key fields such as abstracts, keywords, and references, the total articles included in the analysis was 2063.

### Analysis plan

In recent years, bibliometric analysis has surged in application, driven by advancements in literature databases and computational social sciences^18^, and is now widely used for systematic reviews^19^, identifying research ‘hotspots’^20^, and investigating knowledge production dynamics^21^. Utilizing data mining and visualization technologies, bibliometric analysis provides researchers with a vivid and clear depiction of knowledge development in various fields. This objective and efficient approach enables the analysis of large volumes of academic literature^22^.

Bibliometric analysis objectively interprets massive datasets like citations, abstracts, and keyword frequencies, helping to map and understand the accumulation and evolution of scientific knowledge across fields. Consequently, this study employs bibliometric analysis to focus on the adolescents, exploring the state and characteristics of knowledge production concerning e-cigarettes research. The analysis and visualizations will be facilitated using tools such as Citespace and R package *biblioshiny*, ensuring a comprehensive and insightful exploration of the emergent themes and trends.

Specifically, this study will conduct performance analysis, collaboration network analysis and cocitation network analysis. Performance analysis is a descriptive strategy that assesses the productivity and impact of various journals, countries, institutions, and authors in the field of e-cigarette research among adolescents^22^. In collaboration network analysis, we employ the concept of Betweenness-centrality (BC) to measure the importance different contributors, which represents the number of shortest paths from a given node linking any other two nodes^23^. Additionally, co-citation occurs when two documents are cited together by another document. Analyzing the clustering network of co-cited documents allows us to identify distinct research branches and emerging ‘hotspots’^22^. Additionally, there are other metrics that have been employed to research the current state of adolescents’ e-cigarette use. In order to identify the most prolific authors within the field, we also used a core author formula based on Price’s Law ($M=0.749\sqrt{N_{max}}$), where N_max_ represents the number of publications by the most prolific author in the field^24^. The burst detection refers to keywords that frequently appear in published articles within a short period and is often used to analyze new trends in research activities^25^.

## RESULTS

### Publications and main journals

By tracking the change in the number of published articles, we can intuitively observe the developmental trends of adolescent e-cigarette research over specific periods. Supplementary file Table 1 displays the changes in publication volume from 2002 to 2024. Overall, e-cigarette research literature from 2002 to 2024 demonstrates a significant upward trend, totaling 2063 published articles. In the initial decade, related publications were exceedingly scarce, averaging fewer than two articles per year. Prior to the widespread market popularity of new nicotine products, e-cigarettes received limited research attention and the scientific understanding of their potential health risks was relatively limited. As the health risks associated with e-cigarettes became better understood, the volume of research grew from 25 studies in 2014 to 162 in 2018. Following 2019, the research entered a burst phase, with over 300 articles published annually, peaking at 337 in 2021.

**Table 1:** Top 10 most productive countries, institutions and authors, 2002–2024 (N=2063)

*Country*	*Count*	*BC*	*Institution*	*Count*	*BC*	*Author*	*Count*	*BC*
USA	1477	0.65	University of California System	165	0.14	S. Krishnan-Sarin	61	0.05
Canada	124	0.03	University of Southern California	125	0.07	G. Kong	56	0.1
England	101	0.31	University of Ohio	108	0.21	B. Halpern-Felsher	54	0.03
Australia	75	0.03	University of Texas System	102	0.04	A.M. Leventhal	51	0.02
China	57	0	Yale University	100	0.09	J.L. Barrington-Trimis	48	0.03
South Korea	54	0.12	University of California San Francisco	82	0.01	J.B. Unger	36	0.1
Scotland	40	0.06	Stanford University	71	0.03	M.B. Harrell	35	0.03
Italy	33	0.03	Johns Hopkins University	68	0.14	D.R. Camenga	30	0
Germany	28	0	University of North Carolina	66	0.07	M.E. Morean	25	0
Poland	27	0	University of Michigan	66	0.02	K.W. Bold	25	0

Considering the sources of publication, the selected articles were published across 481 different journals. Supplementary file Table 2 lists the top 10 journals that published the most articles on the topic. Since 2002, *Addictive Behaviors* and *Nicotine & Tobacco Research* have been the leading journals publishing research on adolescent e-cigarette behavior, with 177 (16.1%) and 176 (16.0%) articles, respectively. This is followed by *International Journal of Environmental Research and Public Health* and the *Journal of Adolescent Health*, each having published 122 articles (11.1%).

### Country/Institutional/Author collaboration

Research scholars, along with their institutions and countries, can elucidate the trajectory of intellectual development within collaborative networks, while also providing valuable insights for future research^26^. This section will analyze the global geographical distribution and collaboration networks by showcasing the top ten countries, institutions, and authors with the most contributions to adolescent e-cigarette research (Table 1).

Firstly, the US leads globally with an output of 1477 documents. BC measures a node’s importance and influence within a network, particularly its capacity as a mediator. The BC value of 0.65 for the US also underscores its pivotal role in global collaborations. Following are Canada (N=124), the United Kingdom (N=101), and Australia (N=75), with the United Kingdom noted as the largest international collaborator after the US (BC=0.31). Contributions from outside Europe and North America have increased significantly, with Asian nations like China and South Korea each publishing over 50 articles. South Korea’s BC value of 0.12 highlights its tight collaborative ties within the network.

Similar observations are evident in the institutions that published these articles. The University of California System (N=165), the University of Southern California (N=125), and the University of Ohio (N=108) are the top three institutions in terms of publication volume. The University of Ohio not only leads in quantity but also occupies a significant position in the institutional collaboration network (BC=0.21). Together with Johns Hopkins University (N=68, BC=0.14), it engages in close collaboration globally with other organizations and institutions. Notably, the top ten most productive institutions are located in the US, whose research articles are widely cited and disseminated. This further underscores the leadership of the US in this research field.

From 2002 to 2024, a total of 6718 researchers have published articles in the adolescent e-cigarette use field. Table 1 lists the top ten authors by publication volume, with S. Krishnan-Sarin of Yale University School of Medicine being the most prolific scholar, having published 61 articles over more than two decades. Additionally, the core author analysis revealed that there are 55 core authors in the adolescent e-cigarette use field, each having published more than six articles. Among them, G. Kong (N=56, BC=0.1) and J. B. Unger (N=36, BC=0.1) not only lead in publication quantity but are also key drivers of collaborative research in this field.

### Discipline evolution and collaboration

**Figure 1:**
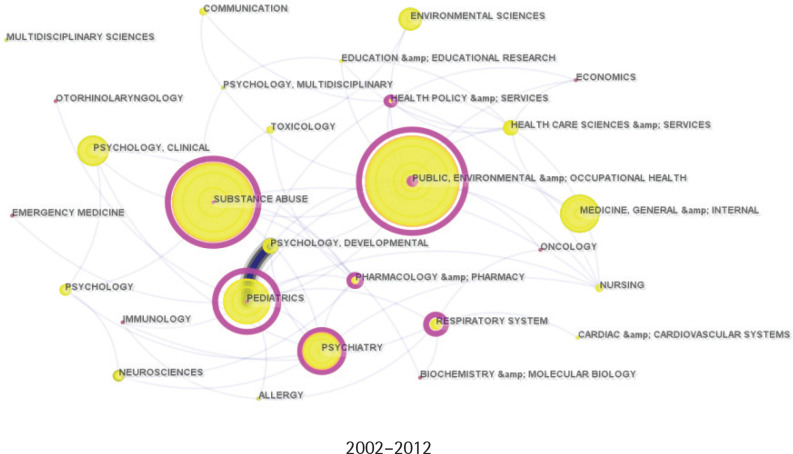
Category collaboration network from 2002 to 2012. Each node indicates a category, and the larger the node, the more articles were published. Each edge indicates a collaborative relationship between two categories (N=5)

**Figure 2:**
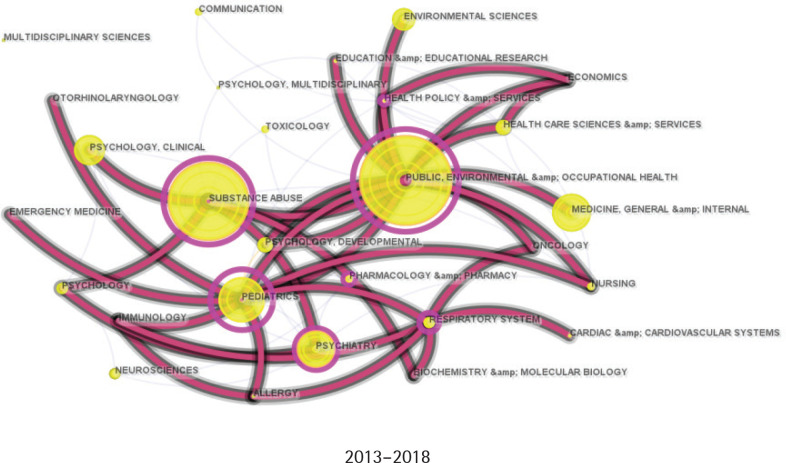
Category collaboration network from 2013 to 2018. Each node indicates a category, and the larger the node, the more articles were published. Each edge indicates a collaborative relationship between two categories (N=468)

**Figure 3:**
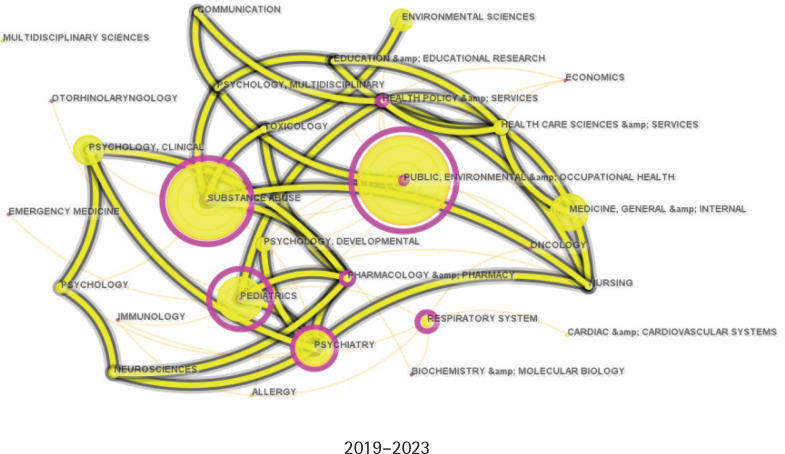
Category collaboration network from 2019 to 2024. Each node indicates a category, and the larger the node, the more articles were published. Each edge indicates a collaborative relationship between two categories (N=1590)

Figures 1–3 illustrate the disciplinary expansion and development within adolescent e-cigarette research. Based on the historical publication data, the field’s evolution is divided into three periods: the initial stage (2002–2012), the development stage (2013–2018), and the prosperity stage (2019–present). Initially, research on adolescent e-cigarettes was primarily concentrated in public health, pediatrics, and psychiatry. After 2013, the research fields expanded significantly, forming a core disciplinary system centered on public health, medicine, and rapidly growing substance abuse, supplemented by psychology, education, and social and environmental sciences. In the past five years, as e-cigarettes have become increasingly popular among adolescents, more disciplines have begun to address this issue, further enhancing the diversity of academic collaborations.

### Research ‘hotspots’ and trends

Keywords summarize the article’s content, closely linked to the research theme and questions, and inherently connected to the current ‘hotspots’ in existing literature. Supplementary file Figure 2 displays the co-occurrence and word cloud, highlighting new trends in adolescent e-cigarette use behavior. In addition to terms directly related to the research topic like e-cigarettes and adolescents, other frequently appearing keywords from 2002 to 2024 include vaping, electronic nicotine delivery system (ENDS), substance use, public policy, prevention, advertising, and cessation. Specifically, knowledge production regarding adolescent e-cigarettes has expanded beyond traditional medical and public health issues.

**Figure 4:**
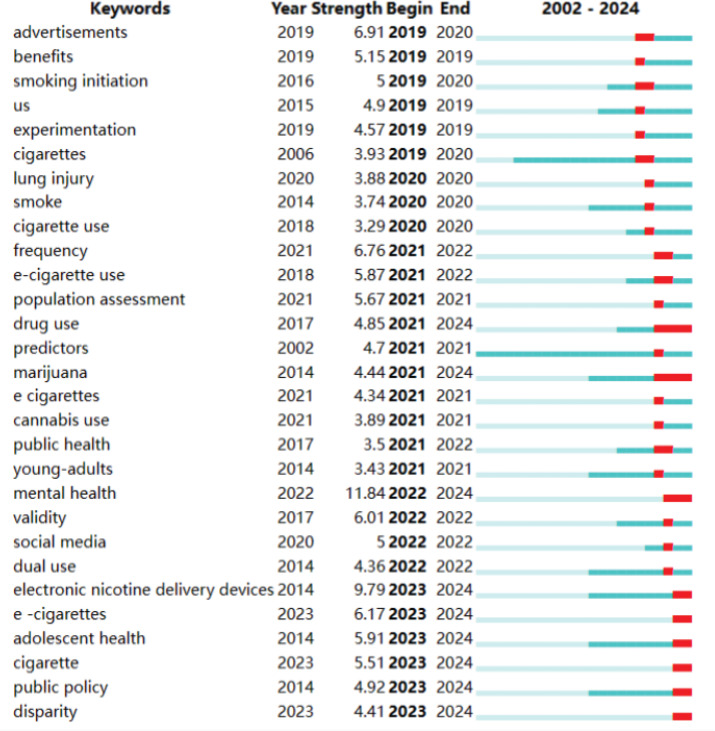
The keywords with the Strongest Citation Bursts. The beginning of a blue line depicts when an article is published. The beginning of a red segment marks the beginning of a period of burst, whereas the end of the red segment marks the end of the burst period (2002–2024) (N=2063)

To better visualize the temporal changes in research ‘hotspots’, an analysis of keyword emergence since 2019 has been included (Figure 4). Keywords like cigarettes and predictors appeared early, but their burst phase lasted only 1–2 years, suggesting that research has shifted to more specific subtopics. Recently, keywords such as advertisement, smoking initiation, lung injury, and social media have garnered significant attention, with their emergence generally spanning from 2020 to 2022. The recent persistence of keywords such as drug use, marijuana, mental health, ENDS, adolescent health, public policy, and disparity into 2024, indicates these areas as emerging research ‘hotspots’.

In addition to keyword analysis, we further explore current trends in adolescent e-cigarette research through the analysis of co-citation clusters. Supplementary file Figure 3 displays the results of the largest 18 clusters. Certain interesting clusters deserve further explanation. Cluster electronic cigarettes (#0) is a foundational cluster focusing on the prevalence, patterns, and immediate health effects of e-cigarette use among adolescents. Cluster (#1) explores the relationship between traditional cigarette smoking and e-cigarette use. Cluster (#2) critically examines how flavors attract young users and the regulatory challenges posed by flavored e-cigarettes. Cluster (#3) evaluates the impact of e-cigarette advertising strategies on shaping adolescents’ perceptions. EVALI (E-cigarette or Vaping Product Use Associated Lung Injury) (#4) highlights association between vaping and the risks of severe lung injuries. Cluster (#6) delves into the motivations for e-cigarette use among adolescents, including social influences and curiosity. Cluster (#10) explores narratives involving personal stories and media depictions that influence perceptions and risk awareness of e-cigarettes among various stakeholders. Cluster (#11) examines behavioral changes in adolescents, such as the transitions between vaping and smoking. Cluster (#12) studies the impact of peer e-cigarette use on adolescents. Cluster (#14) investigates the broader psychosocial factors influencing adolescent e-cigarette use, including socioeconomic status and family dynamics. Cluster (#17) examines the relationship between e-cigarette use and substance use disorders, analyzing how vaping influences or is influenced by other substance use behaviors.

Research on adolescent e-cigarettes, as indicated by cluster labels, can be categorized into several directions: attractiveness (#2, #3), environment and health (#4, #5, #9), accessibility and smoking behavior (#1, #6, #7, #8, #10, #11, #12, #16, #17), and mental health (#13, #14, #15). These four aspects not only showcase the multifaceted nature of this research field but also highlight the need for an interdisciplinary approach to addressing adolescent e-cigarette use. Besides electronic cigarettes (#0), indoor air quality (#5), pregnancy (#7), and tobacco products (#8), most clusters represent topics that are currently and will continue to be actively researched. Emerging trends such as increased drug use, mental health considerations, the impact of public policy, and social disparities run across multiple analysis modules. These interrelated themes create a comprehensive map of the current state of adolescent e-cigarette research, identifying areas that require ongoing focus and enhanced research as market and social contexts evolve.

## DISCUSSION

This study employs bibliometric analysis to explore the knowledge production status and thematic trends in research on adolescent e-cigarette use. Results show that since the publication of the first related article, the volume of research publications in this field has continuously increased. E-cigarettes are rapidly gaining popularity among adolescents worldwide^1,27^. It is undeniable that discussions in academic journals about this topic are increasing. Despite the urgency of issues related to adolescent e-cigarette use, the scale of existing research remains insufficient. Furthermore, an analysis of academic production and collaboration indicates that Western countries, notably the US, exhibit substantial scholarly output and significant impact in the field of adolescent e-cigarette use, with their researchers and institutions playing vital roles in fostering international and regional cooperation. The extent of research collaboration underscores the trend towards international cooperation in e-cigarette research, critical for shaping global public health policies. In contrast, although developing countries in Africa and India face serious issues with adolescent e-cigarette use, related research and discussions are markedly lacking^28^. Additionally, research on adolescent e-cigarettes is increasingly focusing on broader social science perspectives, beyond single health issues. Collaboration between different disciplinary fields is becoming more intense. For instance, researchers in the field of mental health are beginning to focus on the long-term effects of e-cigarette use on the mental and emotional well-being of adolescents, revealing connections between e-cigarette use and adolescents’ mental health problems such as depression and anxiety. It also emphasizes intervention strategies that enhance adolescents’ awareness of the risks associated with e-cigarette use.

Additionally, the analysis of keywords and co-citation clusters reveals the current hotspots and evolving trends in adolescent e-cigarette research. Recent research ‘hotspots’ have focused on vaping products or devices, advertising and marketing, drug or substance use, public policy research, prevention, and cessation. Keywords with the Strongest Citation Bursts also indicate important research topic, including e-cigarettes containing marijuana, novel devices like disposable e-cigarettes and nicotine pouches, adolescent mental health, public policy, and disparity. Findings from co-citation clusters also validate these insights and identify research frontiers. With technological advancements, novel e-cigarette devices that can adjust vapor output or offer multiple flavors, as well as those containing cannabis components like tetrahydrocannabinol (THC), are gaining increased popularity in the market^29^. This trend significantly heightens adolescents’ exposure to health risks associated with nicotine and THC. Consequently, numerous studies have shifted focus to e-cigarette advertising and marketing, exploring how these strategies employ a youthful, fashionable image to attract adolescents and amplify their impact via social media platforms^30^. This accessibility could lower barriers for adolescents to acquire e-cigarettes, potentially leading to increased adverse behaviors and mental health issues^31^. Simultaneously, the importance of formulating and implementing public policies targeting adolescent e-cigarette use is underscored, along with how intervention programs might reduce its prevalence and dependence^32^. Such policies might encompass product sales limits, advertising bans, and regulations on products containing nicotine and other ingredients, consistent with global tightening of e-cigarette market regulations, including new laws in countries like the UK and Australia^33^. Furthermore, some research addresses socioeconomic disparities in adolescent e-cigarette use and health outcomes, finding that adolescents from economically disadvantaged or educationally underserved communities are more likely to access and use e-cigarettes^34^. Understanding these disparities can offer crucial insights for policy engagement and interventions aimed at reducing adolescent e-cigarette use.

### Limitations

This bibliometric study has some limitations. First, the search was limited to a specific language and document types. Future studies could expand collaboration with researchers from various countries and linguistic backgrounds, and include conference articles and books. Secondly, it is important to note that this study exclusively utilized the WOS Core Collection database, which may not encompass all research on youth e-cigarette use. Future studies should consider integrating additional databases to obtain a more comprehensive literature perspective. Additionally, the bibliometric analysis was limited to evaluating representative literature that had been published, making it challenging to objectively assess recently published high-quality articles. These articles will also require our continued attention.

## CONCLUSIONS

This study focuses on more than two decades of research concerning adolescent e-cigarette use. Employing bibliometric methods and tools, we analyzed all articles and reviews published in this field within the Web of Science, revealing the growth trajectory of publications and key journals involved. Additionally, we identified the countries, institutions, and authors that have contributed significantly to adolescent e-cigarette research, illustrating the evolution of interdisciplinary cooperation across three distinct periods. Through combined keyword and co-citation cluster analysis, we explored current ‘hot topics’ and emerging trends in this research area. The findings highlight the complexity and interdisciplinary nature of adolescent e-cigarette use, providing researchers with a comprehensive overview of the field via knowledge maps.

## Supplementary Material



## Data Availability

The data supporting this research are available from the authors on reasonable request.
