# Supplementary material for: Trends of electronic cigarette use among adolescents: A bibliometric analysis
Source: Tob Induc Dis. 2024 Aug 22;22:10.18332/tid/191761. doi: 10.18332/tid/191761 (PMC11339947; doi:10.18332/tid/191761)
Supplement: Supplementary file 1 [file TID-22-146-s1.pdf]

Supplementary

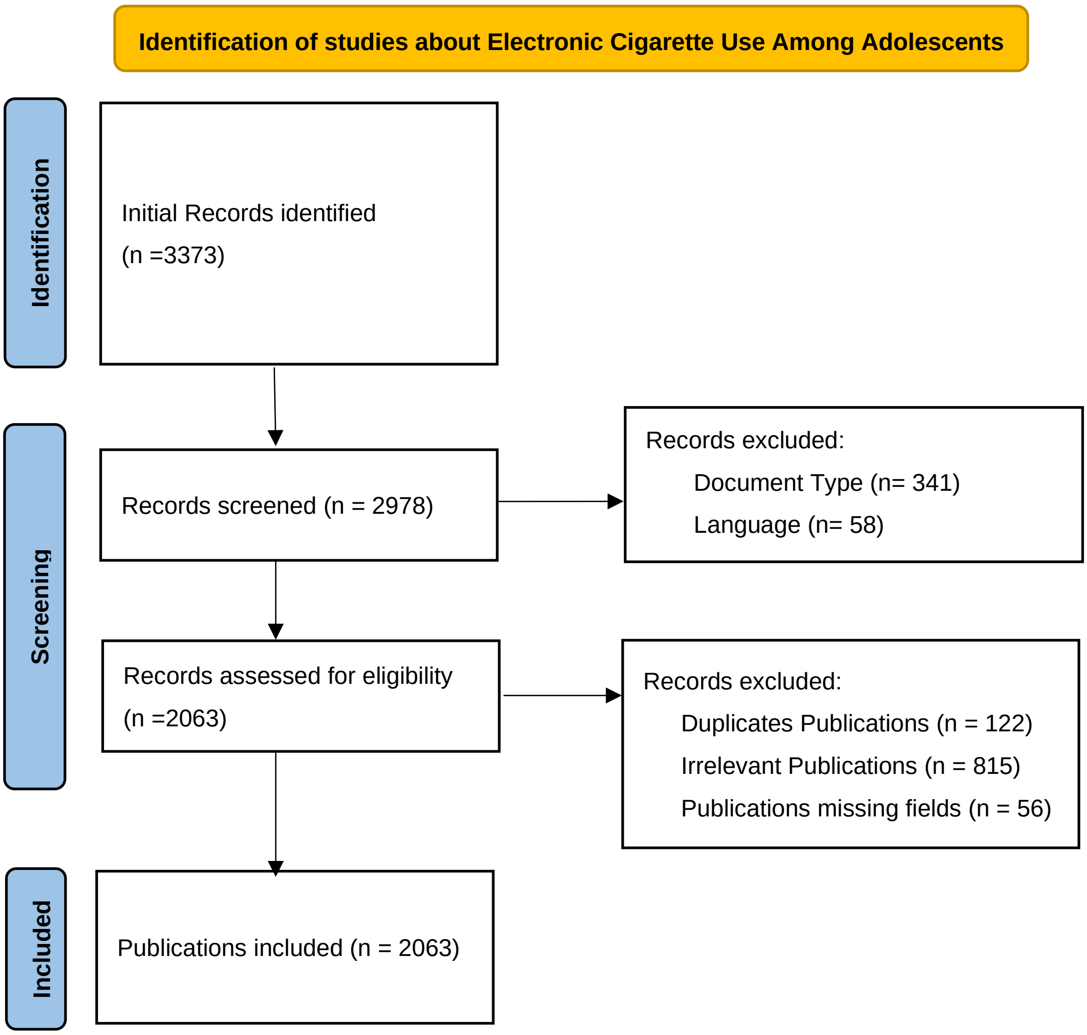

Supplementary Figure 1. *The Preferred Reporting Items for Systematic Reviews (PRISMA) flow diagram*

Supplementary Table 1. *Global annual trend of publications (2002-2024) (N=2063)*

Year	Count	%
2002	1	0.05%
2006	1	0.05%
2011	1	0.05%
2012	2	0.10%
2013	3	0.15%
2014	25	1.21%
2015	69	3.34%
2016	94	4.56%
2017	115	5.57%
2018	162	7.85%
2019	235	11.39%
2020	301	14.59%
2021	337	16.34%
2022	319	15.46%
2023	322	15.61%
2024	76	3.68%

Supplementary Table 2. *Top 10 most productive journals of PYD (2002-2024) (N=2063)*

Journal	Count	%	IF (2022)
Addictive Behaviors	177	16.09%	3.7
Nicotine & Tobacco Research	176	16.00%	3.0
International Journal of Environmental Research and Public Health	122	11.09%	4.6
Journal of Adolescent Health	122	11.09%	5.5
Drug and Alcohol Dependence	106	9.64%	3.9
Tobacco Control	106	9.64%	4.0
Pediatrics	82	7.45%	6.2
Substance Use & Misuse	75	6.82%	1.8
Preventive Medicine	71	6.45%	4.3
BMC Public Health	63	5.73%	3.5

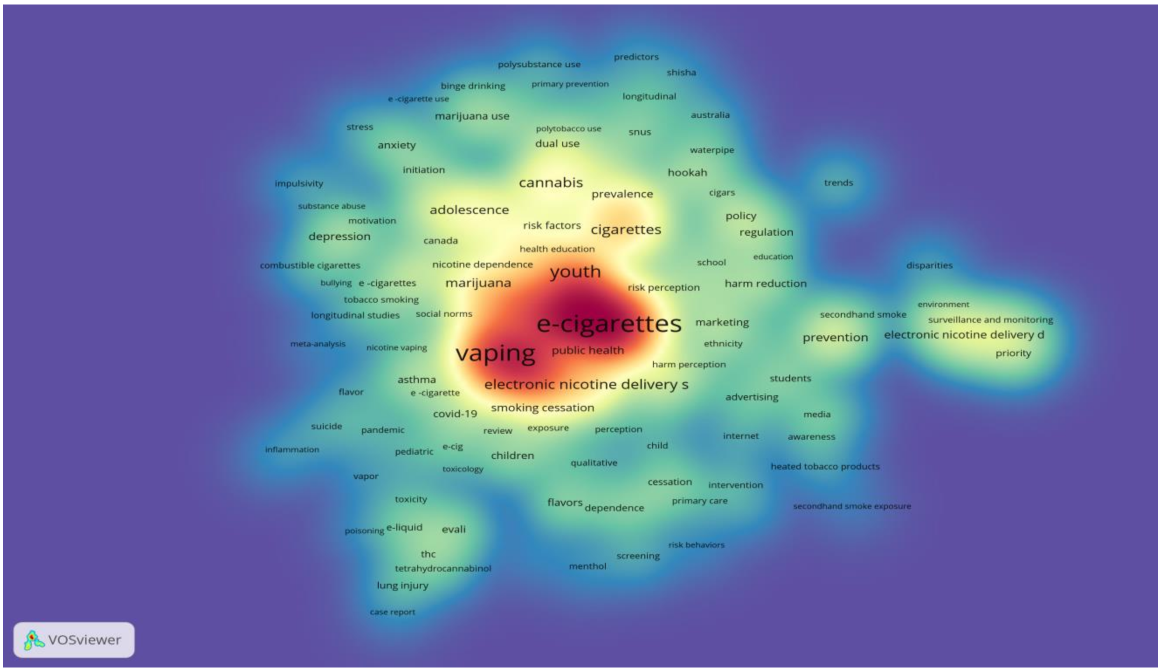

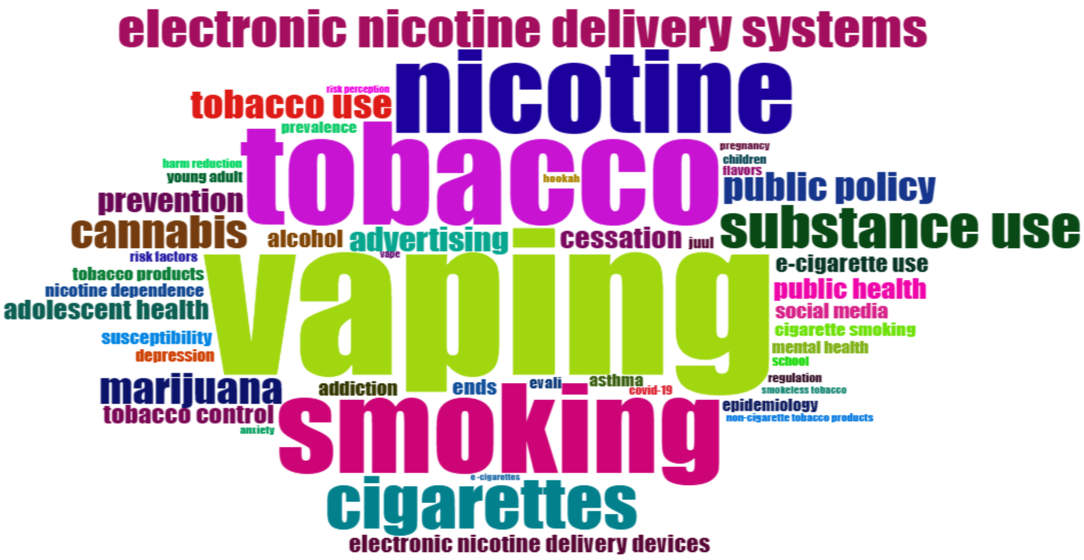

Supplementary Figure 2. *Word-clouds by keywords and keyword co-occurrence network. The font size and colour of a keyword indicates how often it appears, and the larger the font size and darker the colour, the more often it appears. (2002-2024) (N=2063)*

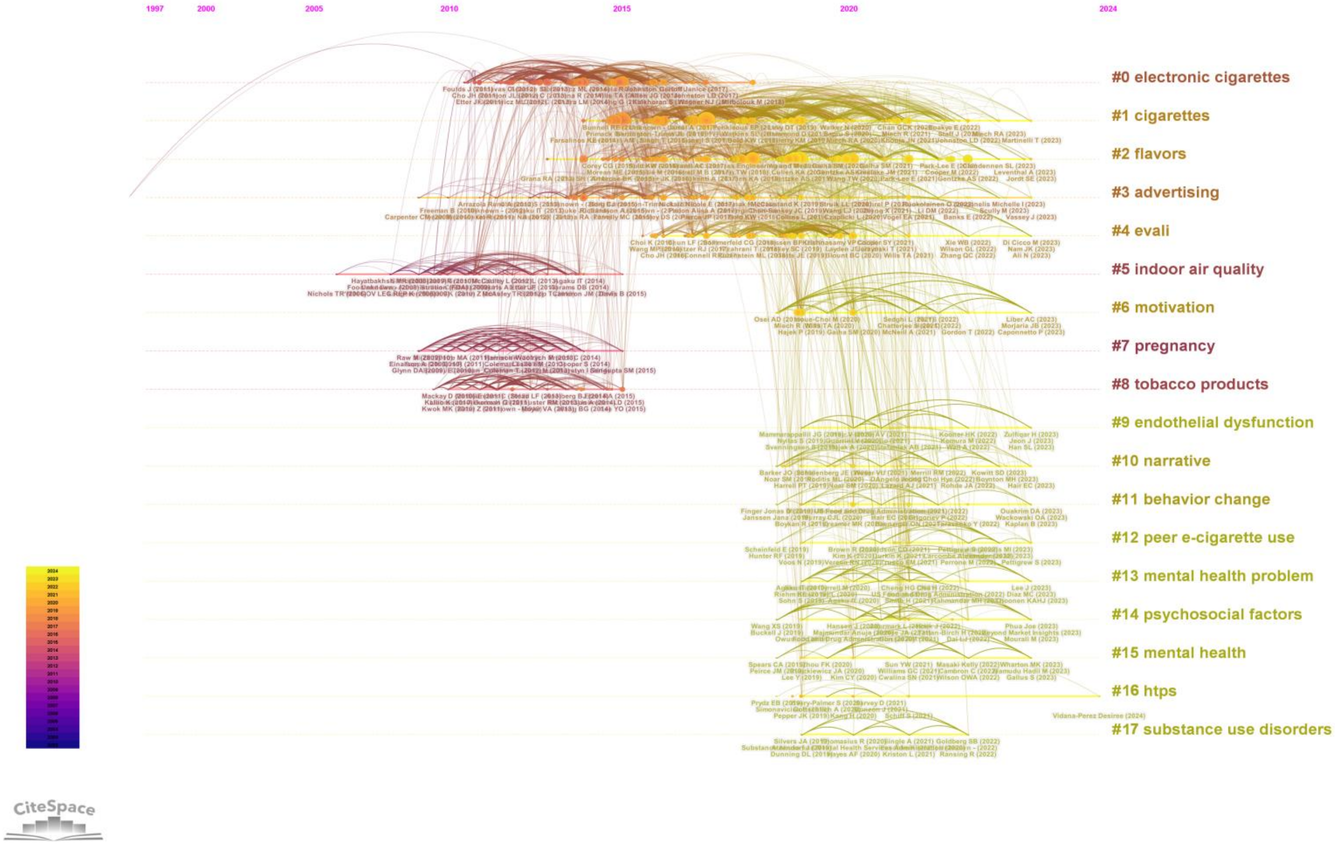

Supplementary Figure 3. *Timeline view of co-citation clusters. The timeline visualizes the largest 18 clusters of cited literature along the horizontal timeline. Each cluster is arranged vertically in descending order of size, with nodes within the cluster arranged chronologically on the same horizontal line. Colored curves represent co-citation links added in the corresponding colored years. The closer the nodes are to the right, the more recent the topic of a cluster. (2002-2024) (N=2063)*

©2024 Chen W. et al.
